# Microbial Succession and Flavor Production in the Fermented Dairy Beverage Kefir

**DOI:** 10.1128/mSystems.00052-16

**Published:** 2016-10-04

**Authors:** Aaron M. Walsh, Fiona Crispie, Kieran Kilcawley, Orla O’Sullivan, Maurice G. O’Sullivan, Marcus J. Claesson, Paul D. Cotter

**Affiliations:** a Teagasc Food Research Centre, Moorepark, Fermoy, Co. Cork, Ireland; b APC Microbiome Institute, University College Cork, Co. Cork, Ireland; c Microbiology Department, University College Cork, Co. Cork, Ireland; d School of Food and Nutritional Sciences, University College Cork, Co. Cork, Ireland; University of California, San Diego

**Keywords:** dairy, flavor, kefir, metagenomics, microbiota

## Abstract

Kefir is a putatively health-promoting dairy beverage that is produced when a kefir grain, consisting of a consortium of microorganisms, is added to milk to initiate a natural fermentation. Here, a detailed analysis was carried out to determine how the microbial population, gene content, and flavor of three kefirs from distinct geographic locations change over the course of 24-h fermentations. Metagenomic sequencing revealed that *Lactobacillus kefiranofaciens* was the dominant bacterial species in kefir during early stages of fermentations but that *Leuconostoc mesenteroides* became more prevalent in later stages. This pattern is consistent with an observation that genes involved in aromatic amino acid biosynthesis were absent from *L. kefiranofaciens* but were present in *L. mesenteroides*. Additionally, these shifts in the microbial community structure, and associated pathways, corresponded to changes in the levels of volatile compounds. Specifically, *Acetobacter* spp. correlated with acetic acid; *Lactobacillus* spp. correlated with carboxylic acids, esters and ketones; *Leuconostoc* spp. correlated with acetic acid and 2,3-butanedione; and *Saccharomyces* spp. correlated with esters. The correlation data suggest a causal relationship between microbial taxa and flavor that is supported by observations that addition of *L. kefiranofaciens* NCFB 2797 increased the levels of esters and ketones whereas addition of *L. mesenteroides* 213M0 increased the levels of acetic acid and 2,3-butanedione. Finally, we detected genes associated with probiotic functionalities in the kefir microbiome. Our results illustrate the dynamic nature of kefir fermentations and microbial succession patterns therein and can be applied to optimize the fermentation processes, flavors, and health-related attributes of this and other fermented foods.

**IMPORTANCE** Traditional fermented foods represent relatively low-complexity microbial environments that can be used as model microbial communities to understand how microbes interact in natural environments. Our results illustrate the dynamic nature of kefir fermentations and microbial succession patterns therein. In the process, the link between individual species, and associated pathways, with flavor compounds is revealed and several genes that could be responsible for the purported gut health-associated benefits of consuming kefir are identified. Ultimately, in addition to providing an important fundamental insight into microbial interactions, this information can be applied to optimize the fermentation processes, flavors, and health-related attributes of this and other fermented foods.

**Author Video**: An author video summary of this article is available.

## Abstract (precis)

Traditional fermented foods represent relatively low-complexity microbial environments that can be used as model microbial communities to understand how microbes interact in natural environments. Our results illustrate the dynamic nature of kefir fermentations and microbial succession patterns therein. In the process, the link between individual species, and associated pathways, with flavor compounds is revealed and several genes that could be responsible for the purported gut health-associated benefits of consuming kefir are identified. Ultimately, in addition to providing an important fundamental insight into microbial interactions, this information can be applied to optimize the fermentation processes, flavors, and health-related attributes of this and other fermented foods.

## INTRODUCTION

Our knowledge of the composition of complex microbial communities from different environments has increased dramatically in recent years (1–3). However, considerably less is known about the biological interactions and other processes that drive microbial succession, or changes in the microbial population structure over time, in these environments (4). It has been proposed that microbial communities from fermented foods could provide a useful model for elucidating the determinants of microbial succession, given that they are considerably less complex than, for example, those from the gut or soil (5). Indeed, cheese rind communities have previously been used to great effect for this purpose (6).

Here, we show that kefir provides an alternative model microbial community that is less complex and provides results even more quickly. Kefir is a traditional fermented milk beverage that is typically produced by inoculating a kefir grain, a cauliflower-like exopolysaccharide (EPS) matrix containing a symbiotic community of bacteria and yeast (7), into milk and incubating it at room temperature for approximately 24 h, resulting in a beverage that has been described as having a pleasantly sour or yogurt-like taste (8). This flavor can vary, depending on the microbial composition of the grain that is used (9). High-throughput sequencing investigations have demonstrated that kefir grains are typically dominated by the bacterial genus *Lactobacillus* and the fungal phylum *Ascomycota* (9, 10). In contrast, kefir milk is dominated by the bacterial genera *Lactobacillus*, *Lactococcus*, *Acetobacter*, and *Leuconostoc* and the fungal genera *Kazachstania*, *Kluyveromyces*, *Naumovozyma*, and *Saccharomyces* (9, 11, 12).

The consumption of kefir has been associated with numerous health benefits, including anticarcinogenic, anti-inflammatory, and antipathogenic effects (13–15), as well as the alleviation of the symptoms of lactose intolerance and the reduction of cholesterol (16, 17). There is mounting evidence to suggest that the microorganisms present in kefir exert at least some of these health benefits (18–22), but there is a lack of understanding of the mechanisms by which they do so.

In this work, amplicon sequencing and whole-metagenome shotgun sequencing were combined with metabolomics and flavor analysis to highlight how the microbial composition, gene content, and flavor of kefir change over the course of 24-h fermentations. We demonstrate that the integration of multiple omics data can predict the contribution of individual microorganisms to metabolite production in a microbial environment, using flavor formation as an example, and we validate these findings through supplementation with specific microbes. To our knowledge, this is the first study to combine metagenome binning and metabolic reconstruction to determine the microbial composition, at both the species and strain levels, and the functional potential of a fermented food, respectively, at different stages of fermentation. In addition, this is the first study to combine whole-metagenome shotgun sequencing with metabolomics to link microbial species with volatile-compound production in kefir. Our findings reveal a dynamic flux from *Lactobacillus kefiranofaciens* domination during the early stages of fermentations to *Leuconostoc mesenteroides* domination during the latter stages, establish a causal relationship between microbial taxa and flavor, and highlight genes that likely contribute to kefir’s purported health-associated attributes.

## RESULTS

### Microbial composition of kefir.

16S rRNA and internal transcribed spacer (ITS) gene sequencing was used to determine the changes in the microbial population of kefirs over the course of 24-h fermentations initiated with three separate grains, designated Fr1, Ick, and UK3, from distinct geographic locations, namely, France, Ireland, and the United Kingdom.

Analysis of the grains showed that *Lactobacillus* was the dominant bacterial genus and constituted >92% of the populations of all three grains (see Fig. S1 in the supplemental material). *Acetobacter* was subdominant and accounted for between 1 and 2% of the population of each grain. In addition, *Leuconostoc* was present in all three grains, although its abundance varied from 0.2 to 1.5%. Other genera that were detected at a relative abundance of >1% were *Propionibacterium*, in Fr1 (4.6%) only, and *Bifidobacterium*, in UK3 (3.4%) only. A fungal population was detected in the grains Fr1 and Ick but not in UK3. *Saccharomyces* and *Kazachstania* were the only fungal genera present (see Fig. S1).

10.1128/mSystems.00052-16.6 Figure S1  The bacterial (A) and fungal (B) compositions of kefir grains, as determined by amplicon sequencing. Note that we were unable to generate an ITS amplicon for the UK3 sample. Download Figure S1, PDF file, 0.09 MB. Copyright © 2016 Walsh et al. 2016 Walsh et al. This content is distributed under the terms of the Creative Commons Attribution 4.0 International license.

**FIG 1:**
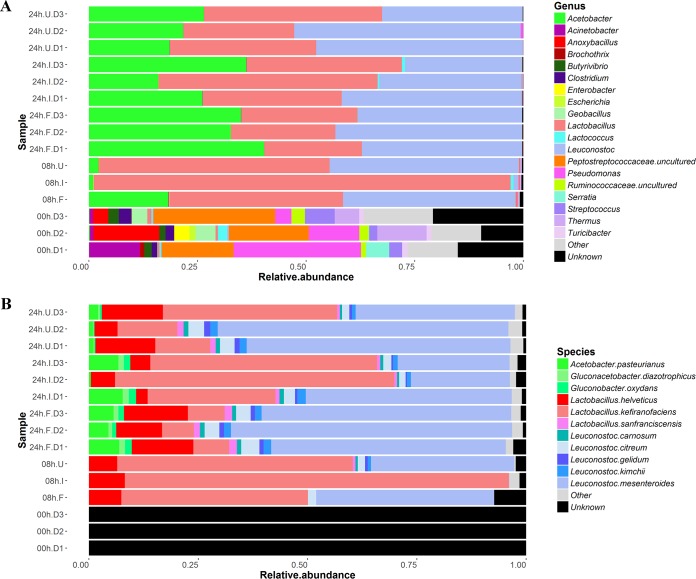
Stacked bar charts presenting the bacterial compositions of kefir samples after 0, 8, and 24 h of fermentation, as determined by 16S rRNA gene sequencing (A) and binning of metagenome sequences with Kraken (B).

Analysis of milk samples revealed that an initially relatively high bacterial diversity decreased over time, with a small number of genera becoming dominant by 8 and 24 h (see Fig. S2 in the supplemental material). On average, at 0 h, or immediately before the grains were added to the milk, the bacterial genera present at a relative abundance of ≥1% were *Pseudomonas* (16.9%), *Anoxybacillus* (7.1%), *Thermus* (6.5%), *Acinetobacter* (5%), *Streptococcus* (4.5%), *Geobacillus* (3.2%), *Clostridium* (2.4%), *Butyrivibrio* (2.2%), *Serratia* (2.1%), *Enterobacter* (1.3%), *Turicibacter* (1.3%), and *Lactococcus* (1%). A further 46.5% of the bacterial genera had a relative abundance of <1% (Fig. 1A). This microbial profile is consistent with that of pasteurized milk, as reported previously by Quigley et al. (23). We were unable to generate an ITS amplicon for the three samples collected at 0 h, and quantitative PCR (qPCR) indicated that fungal DNA was present at <2 pg/µl.

10.1128/mSystems.00052-16.7 Figure S2  Cladogram presenting the bacterial diversity of kefir samples at 0, 8, and 24 h of fermentation, as determined by 16S rRNA gene sequencing. The color intensities in the outer rings indicate the average relative abundances of the bacterial genera in Fr1, Ick, and UK3 at each stage. Download Figure S2, DOCX file, 0.6 MB. Copyright © 2016 Walsh et al. 2016 Walsh et al. This content is distributed under the terms of the Creative Commons Attribution 4.0 International license.

qPCR measurements revealed that the total bacterial and fungal levels increased after kefir grains were added to milk (see Table S1 in the supplemental material). At 8 and 24 h in Fr1, Ick, and UK3, *Lactobacillus*, *Leuconostoc*, and *Acetobacter* accounted for >98% of the total bacterial population, while *Saccharomyces* and *Kazachstania* accounted for >99% of the fungal population. No other bacterial or fungal genera were present at a relative abundance of >1%.

10.1128/mSystems.00052-16.1 Table S1  Absolute abundances of bacteria and fungi in kefir samples after 0, 8, and 24 h of fermentation, as determined by qPCR measurements. Download Table S1, PDF file, 0.06 MB. Copyright © 2016 Walsh et al. 2016 Walsh et al. This content is distributed under the terms of the Creative Commons Attribution 4.0 International license.

Although there were some differences in their compositions at each time point, the bacterial communities of the three kefirs all followed the same pattern of succession (Fig. 1A). Between 0 and 8 h, there was an increase in the relative abundances of *Lactobacillus*, *Leuconostoc*, and *Acetobacter. Lactobacillus* was the dominant genus at 8 h. However, between 8 and 24 h, the relative abundance of *Lactobacillus* decreased. Concurrently, the relative abundances of *Leuconostoc* and *Acetobacter* increased. On average, *Leuconostoc* accounted for approximately one-third of the bacterial population at 24 h. In contrast to the bacterial communities of the three kefirs, the respective fungal communities displayed various patterns of succession (see Fig. S3A in the supplemental material).

10.1128/mSystems.00052-16.8 Figure S3  Stacked bar charts presenting the fungal compositions of kefir samples after 0, 8, and 24 h of fermentation, as determined by ITS gene sequencing (A), and the microbial composition of kefir samples after 0, 8, and 24 h of fermentation (B), as determined by MetaPhlan2. Download Figure S3, TIF file, 0.4 MB. Copyright © 2016 Walsh et al. 2016 Walsh et al. This content is distributed under the terms of the Creative Commons Attribution 4.0 International license.

16S rRNA and ITS compositional data were supplemented by composition-based analysis of shotgun metagenomic data. Kraken (24) was used to determine the bacterial composition of kefir after 0, 8, and 24 h of fermentation and yielded results that corresponded well to amplicon sequencing results at the genus level but which could be further assigned to the species level. It was established that the kefir milk was dominated by *L. kefiranofaciens* at 8 h (Fig. 1B). However, between 8 and 24 h, the relative abundance of *L. kefiranofaciens* decreased, whereas the relative abundance of *Leuconostoc mesenteroides* increased. During the same period, there were also increases in the relative abundances of *Acetobacter pasteurianus*, *Lactobacillus helveticus*, *Leuconostoc citreum*, *Leuconostoc gelidum*, and *Leuconostoc kimchii*. These results were generally consistent with those generated by MetaPhlan2 (25) (see Fig. S3B in the supplemental material), except that MetaPhlan2 did not detect some of the species present in lower abundance (i.e., *A. pasteurianus*, *L. citreum*, *L. gelidum*, or *L. kimchii*). MetaPhlan2 predicted that *Saccharomyces cerevisiae* was the dominant fungal species and that it accounted for 0.9% and 0.2% of the microbiota in kefir at 8 and 24 h of fermentation, respectively. However, it did not detect *Kazachstania* species.

In addition, PanPhlAn (26) was used to provide strain level characterization of the most dominant bacterial species identified by Kraken and MetaPhlan2. The results indicated that, across all of the kefirs tested, the strains present were most closely related to *L. kefiranofaciens* DSM 10550, *L. mesenteroides* ATCC 8293, and *L. helveticus* MTCC 5463 (see Fig. S5 in the supplemental material). Despite this relative homogeneity, it was still apparent that the strains in a particular kefir were more closely related to each other than they were to strains from other kefirs (see Fig. S4 in the supplemental material).

10.1128/mSystems.00052-16.9 Figure S4  Hierarchically clustered heat maps showing the relatedness of *L. kefiranofaciens* (A), *L. mesenteroides* (B), and *L. helveticus* (C) from Fr1, Ick, and UK3 to reference strain genomes, as determined by PanPhlAn. Clustering was done with hclust2 (https://bitbucket.org/nsegata/hclust2). Download Figure S4, TIF file, 0.9 MB. Copyright © 2016 Walsh et al. 2016 Walsh et al. This content is distributed under the terms of the Creative Commons Attribution 4.0 International license.

10.1128/mSystems.00052-16.10 Figure S5  APLSR plot (principal components 1 and 2) of sensory acceptance and RDA data for spiked and unspiked kefir samples. Download Figure S5, PDF file, 0.08 MB. Copyright © 2016 Walsh et al. 2016 Walsh et al. This content is distributed under the terms of the Creative Commons Attribution 4.0 International license.

### Gene content of kefir.

**FIG 2:**
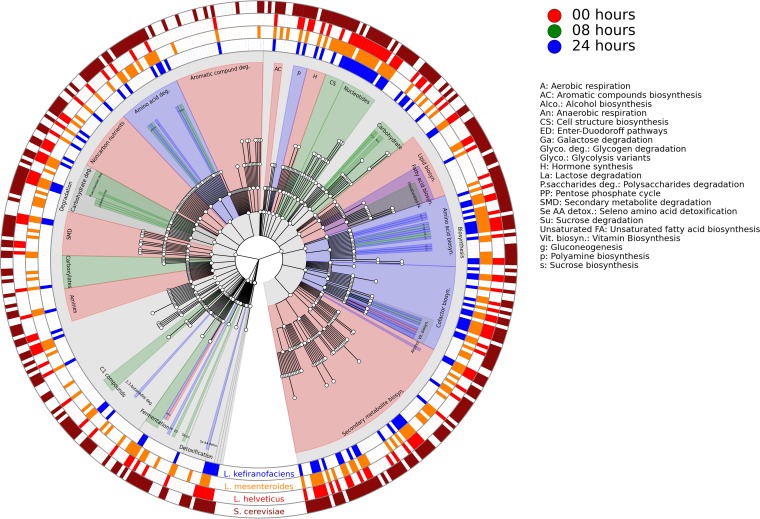
Cladogram presenting a hierarchical overview of the MetaCyc pathways detected in the kefir microbiome with HUMAnN2. Central nodes represent general pathway category functions, like carbohydrate catabolism, and their descendant nodes represent more specific pathway category functions, like sucrose degradation. The colors of the clades indicate the times at which pathways of particular interest were most prevalent, as determined by LEfSe. The outer rings indicate the presence/absence of pathways in *L. kefiranofaciens* (blue), *L. mesenteroides* (orange), *L. helveticus* (red), and *S. cerevisiae* (maroon).

Whole-metagenome shotgun sequencing was used to characterize the functional potential of the kefir microbiome at different stages of fermentation, and the HUMAnN2 pipeline (https://bitbucket.org/biobakery/humann2) was used for metagenomic metabolic reconstruction. The default HUMAnN2 pathway abundance table was regrouped by using a custom mapping file to assign individual MetaCyc pathways (27) to a hierarchy of 534 gene product categories to achieve an overview of the kefir microbiome (Fig. 2). The statistical tool LEfSe (28) was used to identify changes in the abundances of genetic pathways over the course of fermentation. Notably, we observed that pathways involved in carbohydrate metabolism, carboxylate degradation, and unsaturated fatty acid biosynthesis were most prevalent at 8 h, whereas those involved in amino acid metabolism and 2,3-butanediol degradation were most prevalent at 24 h (Fig. 2). Inspection of the default pathway abundance table revealed that pathways involved in fatty acid beta oxidation were present in kefirs. The pathways mentioned here are of particular interest because they are potentially involved in the production of volatile compounds (Table 1).

**TABLE 1:** Volatile compounds detected in kefir by GC-MS

Compound	LRI^a^	Ref LRI^b^	Odor descriptor(s)	Source
Carboxylic acids				
Acetic acid	692	629	Vinegar, peppers, green, fruity, floral, sour	Carbohydrate metabolism
Hexanoic acid	968	983	Sweaty, cheesy, sharp, goaty, bad breath, acidic	Lipid metabolism
Octanoic acid	1,163	1,160	Cheesy, rancid, pungent, sweat, soapy, goaty	Lipid metabolism
Nonanoic acid	1,254	1,276	Fatty, soapy, waxy, green, goat	Lipid metabolism
*n*-Decanoic acid	1,355	1,379	Soapy, waxy, stale, buttery, fruity, grassy, cheesy, milky	Lipid metabolism
Alcohols				
2-Methyl-1-butanol	733	755	Penetrating, alcohol, wine-like, plastic	Amino acid metabolism
2-Ethyl-1-hexanol	1,025	1,031	Animal, cardboard	Lipid metabolism
Ethanol	468	426	Dry, dust	Carbohydrate metabolism
2-Butanol	601	596	Fruity	Carbohydrate metabolism
2-Methyl-1-propanol	621	647	Malty	Amino acid metabolism
3-Methyl-butanol	730	768	Fresh cheese, breathtaking, alcoholic, fruity, grainy, solvent-like, floral, malty	Amino acid metabolism
Phenylethyl alcohol	1,119	1,112	Unclean, rose, violet-like, honey, floral, spicy	Amino acid metabolism
1-Pentanol	730	768	Fruity, alcoholic, green, balsamic, fusel oil, woody	Lipid metabolism
Aldehydes				
3-Methyl-butanal	649	654	Malty, cheesy, green, dark chocolate, cocoa	Amino acid metabolism
2-Methyl-butanal	658	662	Malty, dark chocolate, almond, cocoa, coffee	Amino acid metabolism
Octanal	1,002	1,004	Green, fatty, soapy, fruity, orange peel	Lipid metabolism
Nonanal	1,103	1,106	Green, citrus, fatty, floral	Lipid metabolism
Pentanal	694	697	Pungent, almond-like, chemical, malty, apple, green	Lipid metabolism
Hexanal	798	801	Green, slightly fruity, lemon, herbal, grassy, tallow	Lipid metabolism
Heptanal	900	901	Slightly fruity (balsam), fatty, oily, green, woody	Lipid metabolism
Esters				
Ethyl acetate	609	614	Solvent, pineapple, fruity, apples	Carbohydrate metabolism
Ethyl butanoate	802	800	Ripe fruit, buttery, green, apple, pineapple, banana, sweet	Carbohydrate metabolism
Ethyl hexanoate	995	1,002	Fruity, malty, young cheese, moldy, apple, green, orange, pineapple, banana	Carbohydrate metabolism
Ethyl octanoate	1,190	1,198	Fruity, apple, green, fatty, orange, winey, pineapple, apricot	Carbohydrate metabolism
Ethyl decanoate	1,388	1,396	Fruity, grape, cognac	Carbohydrate metabolism
3-Methyl-1-butanol, acetate	874	879	Fruity, banana, candy, sweet, apple peel	Unknown
Ketones				
2,3-Butanedione	589	596	Buttery, strong	Carbohydrate metabolism
2,3-Pentanedione	694	693	Creamy, cheesy, oily, sweet buttery, caramellic	Carbohydrate metabolism
2,3-Hexanedione	781	788	Sweet, creamy, caramellic, buttery	Carbohydrate metabolism
2-Heptanone	887	891	Blue cheese, spicy, Roquefort	Lipid metabolism
2-Undecanone	1,288	1,294	Floral, fruity, green, musty, tallow	Lipid metabolism
2-Pentanone	679	687	Orange peel, sweet, fruity	Lipid metabolism
2-Nonanone	1,088	1,094	Malty, fruity, hot milk, smoked cheese	Lipid metabolism
Acetone	494	496	Earthy, fruity, wood pulp, hay	Lipid metabolism
2-Butanone	598	593	Buttery, sour milk, etheric	Carbohydrate metabolism
Sulfur compounds				
Dimethyl sulfone	920	926	Sulfurous, hot milk, burned	Amino acid metabolism
Carbon disulfide	537	568	Sweet, ethereal	Amino acid metabolism

a LRI, linear retention index.

b Reference LRI.

In addition, the default HUMAnN2 gene family table was regrouped to Gene Ontology (GO) terms (gene product categories [29]) and, in total, we detected 1,288, 1,006, and 947 GO terms associated with carbohydrate, amino acid, and lipid metabolism in the kefir microbiome. Interestingly, pathways involved in aromatic amino acids and proline biosynthesis were assigned to *L. mesenteroides* but not to *L. kefiranofaciens*. Similarly, pathways involved in arabinose, maltose, pentose, sucrose, xylose, and xylulose metabolism were present in *L. mesenteroides* but not in *L. kefiranofaciens.*

**FIG 3:**
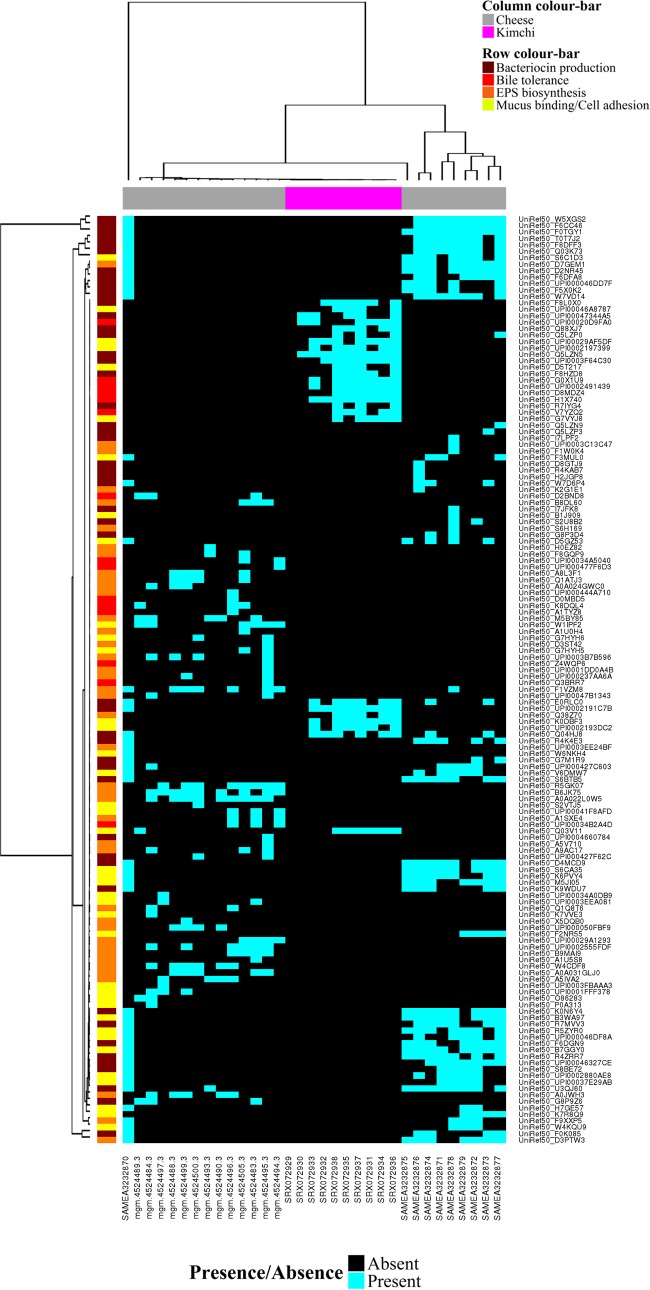
Hierarchically clustered binary heat map showing the presence/absence of genes associated with probiotic action in cheese and kimchi metagenomes, as determined by HUMAnN2. Clustering was performed with the hclust function in the R package gplots.

Finally, the HUMANnN2 gene family table was inspected for genes associated with probiotic functionalities to better understand the basis of the health benefits of kefir. We observed that *L. kefiranofaciens* in Fr1, Ick, and UK3 contained genes encoding EPS synthesis proteins (UniRef50_W5XGS2, UniRef50_F6CC46, and UniRef50_F0TGY1), bile salt transporter proteins (UniRef50_Q74LX5 and UniRef50_F6CE74), adhesion proteins (UniRef50_F6CFB4 and UniRef50_Q040W2), mucus binding proteins (UniRef50_F6CE70, UniRef50_F6CE69, UniRef50_F6CDG7, and UniRef50_F6CBX6), and the type III bacteriocins/bacteriolysins helveticin J (UniRef50_D5GYX2) and enterolysin A (UniRef50_D5GXY3 and UniRef50_F6CAP6). On the basis of these findings, we downloaded publicly available metagenome sequences from cheeses and kimchi (Table 2) todetermine the prevalence of similar genes in other fermented foods. HUMAnN2 indicated that genes encoding EPS synthesis proteins, adhesion proteins, mucus binding proteins, bile salt hydrolases, bile salt symporters, and bacteriocins/prebacteriocins were widespread in the 14 cheese varieties investigated (Fig. 3). In addition, we observed several instances where multiple genes were assigned to individual species (see Table S2 in the supplemental material). We identified similar genes in kimchi (Fig. 5), although HUMAnN2 was unable to assign them to individual species because of the lower sequencing depth of those samples.

**TABLE 2:** Accession numbers of the cheese and kimchi metagenomes analyzed in this study

Origin	Accession no.	Repository	Sample description	Reference
Cheese	4524483.3	MG-Rast	Washed unpasteurized cow cheese	6
Cheese	4524484.3	MG-Rast	Bloomy unpasteurized goat cheese	6
Cheese	4524488.3	MG-Rast	Natural unpasteurized cow cheese	6
Cheese	4524489.3	MG-Rast	Bloomy unpasteurized goat cheese	6
Cheese	4524490.3	MG-Rast	Natural unpasteurized cow cheese	6
Cheese	4524493.3	MG-Rast	Natural unpasteurized cow cheese	6
Cheese	4524494.3	MG-Rast	Washed pasteurized cow cheese	6
Cheese	4524495.3	MG-Rast	Washed unpasteurized cow cheese	6
Cheese	4524496.3	MG-Rast	Washed unpasteurized cow cheese	6
Cheese	4524497.3	MG-Rast	Natural pasteurized cow cheese	6
Cheese	4524499.3	MG-Rast	Washed unpasteurized cow cheese	6
Cheese	4524500.3	MG-Rast	Washed unpasteurized cow cheese	6
Cheese	4524505.3	MG-Rast	Washed unpasteurized cow cheese	6
Kimchi	SRX072929	Sequence Read Archive	Kimchi fermentation, day 1	41
Kimchi	SRX072930	Sequence Read Archive	Kimchi fermentation, day 7	41
Kimchi	SRX072931	Sequence Read Archive	Kimchi fermentation, day 13	41
Kimchi	SRX072932	Sequence Read Archive	Kimchi fermentation, day 16	41
Kimchi	SRX072933	Sequence Read Archive	Kimchi fermentation, day 18	41
Kimchi	SRX072934	Sequence Read Archive	Kimchi fermentation, day 21	41
Kimchi	SRX072935	Sequence Read Archive	Kimchi fermentation, day 23	41
Kimchi	SRX072936	Sequence Read Archive	Kimchi fermentation, day 25	41
Kimchi	SRX072937	Sequence Read Archive	Kimchi fermentation, day 27	41
Kimchi	SRX072938	Sequence Read Archive	Kimchi fermentation, day 29	41
Cheese	SAMEA3232870	European Nucleotide Archive	Continental-type cheese	23
Cheese	SAMEA3232871	European Nucleotide Archive	Continental-type cheese	23
Cheese	SAMEA3232872	European Nucleotide Archive	Continental-type cheese	23
Cheese	SAMEA3232873	European Nucleotide Archive	Continental-type cheese	23
Cheese	SAMEA3232874	European Nucleotide Archive	Continental-type cheese	23
Cheese	SAMEA3232875	European Nucleotide Archive	Continental-type cheese	23
Cheese	SAMEA3232876	European Nucleotide Archive	Continental-type cheese	23
Cheese	SAMEA3232877	European Nucleotide Archive	Continental-type cheese	23
Cheese	SAMEA3232878	European Nucleotide Archive	Continental-type cheese	23
Cheese	SAMEA3232879	European Nucleotide Archive	Continental-type cheese	23

10.1128/mSystems.00052-16.2 Table S2  Microbial species from cheese samples that contain two or more genes associated with probiotic action, as determined by HUMAnN2. Download Table S2, XLSX file, 0.01 MB. Copyright © 2016 Walsh et al. 2016 Walsh et al. This content is distributed under the terms of the Creative Commons Attribution 4.0 International license.

### Volatile-compound profiling and sensory analysis of kefir milk.

**FIG 4:**
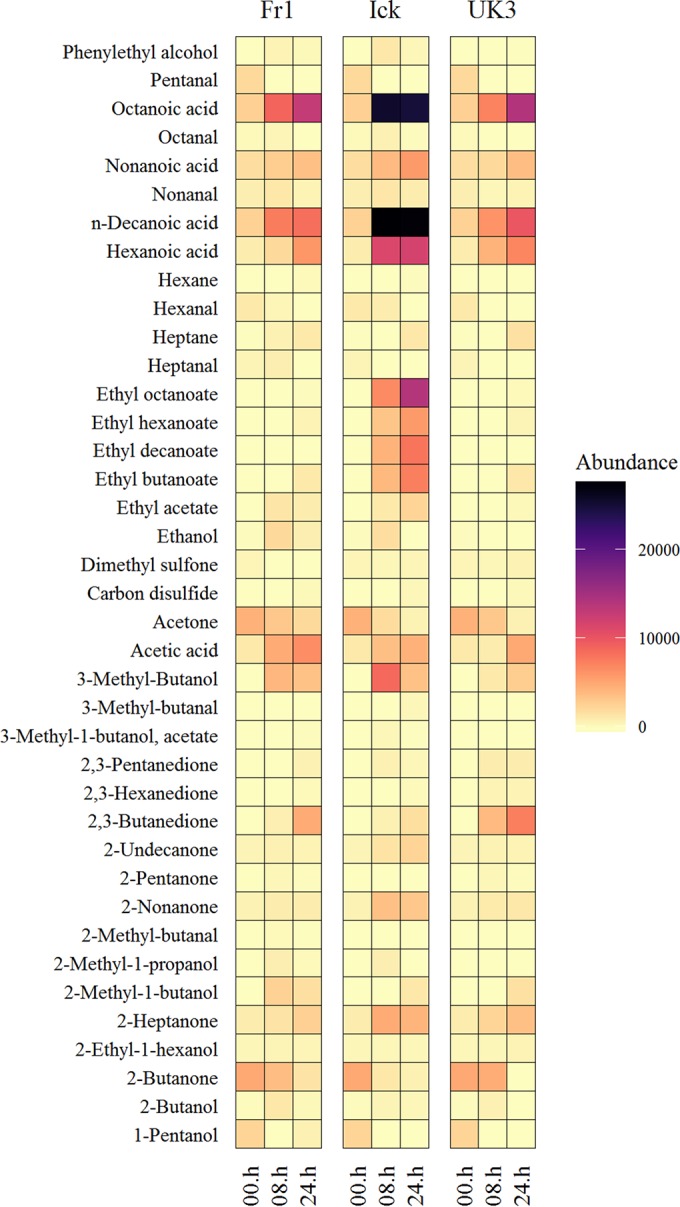
Faceted heat map showing changes in the volatile-compound profiles of Fr1, Ick, and UK3. Note that the 00.h column in each section refers to the volatile-compound profile of milk immediately before fermentation initiation. The same starting milk was used for Fr1, Ick, and UK3.

Gas chromatography-mass spectrometry (GC-MS) was used to determine the volatile-compound profile of kefir milk after 0, 8, and 24 h of fermentation. Thirty-nine volatile compounds that could contribute to flavor were identified and semiquantified in kefir milks produced with each of the three kefir grains. These consisted of nine ketones, seven aldehydes, six esters, eight alcohols, five carboxylic acids, and two sulfur compounds (Table 1). The results of the volatile-compound analysis are presented in Fig. 4. The levels of all of the compounds detected increased after 0 h, apart from 1-pentanol, pentanal, hexanal, heptanal, heptanol, acetone, and 2-butanone (Fig. 4).

Sensory acceptance evaluation and ranking descriptive analysis (RDA) of the Fr1 and Ick kefir milks were performed after 24-h fermentations. These revealed perceptible differences between the milks. Specifically, Fr1 samples had a more likeable, buttery flavor whereas Ick samples had a less likeable but fruity flavor (see Fig. S5 in the supplemental material). These results confirm that the volatile-compound profile data are consistent with subsequent flavor.

### Correlations between microbial taxa and volatile compounds.

**FIG 5:**
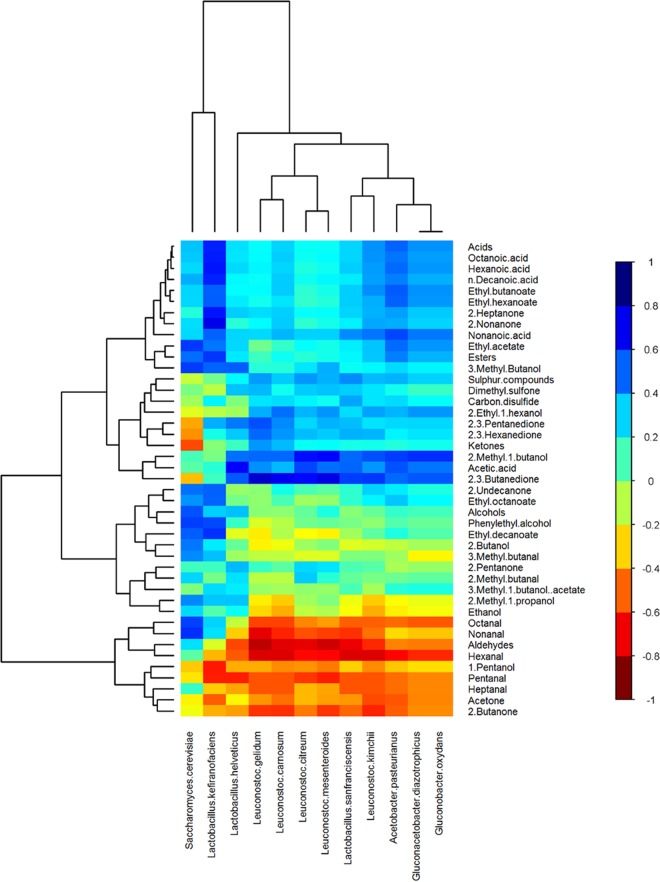
Hierarchically clustered heat map showing correlations between the relative abundances of microbial species and the levels of volatile compounds in kefir samples. Clustering was performed with the hclust function in the R package gplots. The color of each tile of the heat map indicates the type/strength of the correlation for a given species-compound combination, as indicated by the color key.

The Spearman rank correlation test was used to identify correlations between the levels of individual taxa and flavor compounds. At the genus level, based on amplicon sequencing results, there were strong correlations between *Lactobacillus* and carboxylic acids, esters, and 3-methyl-1-butanol; between *Saccharomyces* and carboxylic acids and esters; between *Acetobacter* and acetic acid, 2-methyl-1-butanol, and 2,3-butanedione; between *Leuconostoc* and 2,3-butanedione; and between *Kazachstania* and acetic acid, 2-methyl-1-butanol, 2,3-butanedione, 2,3-pentanedione, and 2,3-hexanedione (see Table S3 in the supplemental material). At the bacterial species level, on the basis of Kraken results, there were strong correlations between *L. kefiranofaciens* and carboxylic acids and esters; between *A. pasteurianus* and carboxylic acids and 2,3-butanedione; and between *L. mesenteroides* and 2,3-butanedione. At the fungal species level, on the basis of MetaPhlan2 results, there were strong correlations between *S. cerevisiae* and alcohols and esters (Table 3; Fig. 5). In summary, correlations were found between compounds associated with vinegary flavors and *A. pasteurianus*, those associated with cheesy flavors and *L. kefiranofaciens*, those associated with buttery flavors and *L. mesenteroides*, and those associated with fruity flavors and *L. kefiranofaciens* and *S. cerevisiae.*

10.1128/mSystems.00052-16.3 Table S3  Correlations between the relative abundances of microbial genera and the levels of volatile compounds. Download Table S3, DOCX file, 0.01 MB. Copyright © 2016 Walsh et al. 2016 Walsh et al. This content is distributed under the terms of the Creative Commons Attribution 4.0 International license.

**TABLE 3:** Summary of strong positive correlations identified between the relative abundances of species and the levels of metabolites in kefir

Species	Compound(s)	*R* value	*P* value	
			Unadjusted	FDR adjusted^a^
*Leuconostoc mesenteroides*	2,3-Butanedione	0.79	0.0005	0.011
*Lactobacillus kefiranofaciens*	2-Nonanone	0.79	0.0005	0.011
*Lactobacillus helveticus*	Acetic acid	0.75	0.0013	0.017
*Leuconostoc mesenteroides*	2-Methyl-1-butanol	0.74	0.0015	0.017
*Lactobacillus kefiranofaciens*	Hexanoic acid	0.71	0.0033	0.024
*Lactobacillus kefiranofaciens*	2-heptanone	0.71	0.0033	0.024
*Lactobacillus kefiranofaciens*	Octanoic acid	0.70	0.0040	0.024
*Lactobacillus kefiranofaciens*	Acids	0.68	0.0049	0.024
*Lactobacillus kefiranofaciens*	*n*-Decanoic acid	0.68	0.0049	0.024
*Saccharomyces cerevisiae*	Nonanal	0.66	0.0080	0.035
*Lactobacillus kefiranofaciens*	Ethyl decanoate	0.65	0.0089	0.035
*Lactobacillus kefiranofaciens*	Esters	0.64	0.0099	0.035
*Saccharomyces cerevisiae*	Ethyl acetate	0.63	0.0110	0.035
*Saccharomyces cerevisiae*	3-Methyl-butanol	0.63	0.0118	0.035
*Acetobacter pasteurianus*	2-Methyl-1-butanol	0.63	0.0126	0.035
*Saccharomyces cerevisiae*	Phenylethyl alcohol	0.62	0.0134	0.035
*Saccharomyces cerevisiae*	Octanal	0.62	0.0141	0.035
*Acetobacter pasteurianus*	Nonanoic acid	0.60	0.0169	0.040
*Lactobacillus kefiranofaciens*	Phenylethyl alcohol	0.60	0.0179	0.040
*Saccharomyces cerevisiae*	Alcohols	0.59	0.0203	0.040
*Acetobacter pasteurianus*	Acetic acid	0.59	0.0206	0.040
*Lactobacillus helveticus*	2,3-Butanedione	0.57	0.0255	0.040
*Acetobacter pasteurianus*	Ethyl butanoate	0.57	0.0268	0.040
*Lactobacillus kefiranofaciens*	Ethyl hexanoate	0.57	0.0279	0.040
*Lactobacillus helveticus*	3-Methyl-butanol	0.56	0.0283	0.040
*Saccharomyces cerevisiae*	Ethyl decanoate	0.56	0.0283	0.040
*Lactobacillus kefiranofaciens*	Ethyl butanoate	0.56	0.0292	0.040
*Acetobacter pasteurianus*	Ethyl hexanoate	0.56	0.0314	0.040
*Lactobacillus kefiranofaciens*	Nonanoic acid	0.56	0.0315	0.040
*Lactobacillus kefiranofaciens*	2-Undecanone	0.56	0.0315	0.040
*Lactobacillus kefiranofaciens*	Ethyl octanoate	0.55	0.0321	0.040
*Lactobacillus kefiranofaciens*	3-Methyl-butanol	0.55	0.0321	0.040
*Acetobacter pasteurianus*	Acids	0.55	0.0324	0.040
*Acetobacter pasteurianus*	Hexanoic acid	0.54	0.0368	0.044
*Acetobacter pasteurianus*	2,3-Butanedione	0.54	0.0375	0.044
*Acetobacter pasteurianus*	Ethyl acetate	0.54	0.0381	0.044
*Saccharomyces cerevisiae*	Esters	0.53	0.0413	0.046
*Leuconostoc mesenteroides*	Acetic acid	0.53	0.0419	0.046
*Lactobacillus helveticus*	2-Methyl-1-butanol	0.53	0.0433	0.046
*Saccharomyces cerevisiae*	2-Undecanone	0.53	0.0435	0.046
*Lactobacillus kefiranofaciens*	Ethyl acetate	0.52	0.0478	0.048
*Acetobacter pasteurianus*	Octanoic acid	0.52	0.0478	0.048

a FDR, false discovery rate.

### Impact of supplementing kefir with kefir isolates.

The consequences of adding *L. kefiranofaciens* NCFB 2797 to Fr1, a kefir with a low indigenous *L. kefiranofaciens* population level, was investigated. GC-MS revealed that this addition caused increases in the levels of the esters ethenyl acetate (by 59.15%), ethyl acetate (100%), methyl-3-butyrate (26.83%), and 2-methylbutyl-acetate (11.44%) and the ketone 2-heptanone (65.86%). In contrast, the addition of *L. mesenteroides* 213M0 to Ick, a kefir with a low indigenous *L. mesenteroides* population level, resulted in increases in the levels of acetic acid (168.28%) and 2,3-butanediol (14.91%), a precursor to 2,3-butanedione (see Table S4 in the supplemental material). Despite changes in the volatile-compound profile, there were no perceptible changes in flavor (see Fig. S5 in the supplemental material).

10.1128/mSystems.00052-16.4 Table S4  Changes in the volatile-compound profiles of kefirs supplemented with *L. kefiranofaciens* 484 NCFB 2797 (A) and *L. mesenteroides* 213M0 (B). Download Table S4, XLSX file, 0.01 MB. Copyright © 2016 Walsh et al. 2016 Walsh et al. This content is distributed under the terms of the Creative Commons Attribution 4.0 International license.

## DISCUSSION

Many traditional fermented foods have been reported to have health benefits (30, 31). These foods are often produced on a small-scale, artisanal basis. However, the increased demand for health-promoting foods among the public presents an opportunity to bring traditional fermented foods to a wider audience and serves as an incentive to optimize starter cultures for the mass production of fermented foods with enhanced sensory qualities (32). In recent years, genetic characterization has been increasingly employed to guide starter culture development for numerous fermented foods, including wines, beers, cocoa, and meats (33–36). Similarly, integrated molecular omics approaches (37) have emerged as powerful methods of investigating the microbial dynamics of food fermentations with the aim of optimizing processes like flavor production (38). In this study, we combined compositional and shotgun DNA sequencing with GC-MS and flavor analysis to predict microbes involved in the production of different flavor compounds in kefir.

We identified significant correlations between the abundances of particular microbial genera and species and the levels of different volatile compounds and showed that the microbes in kefir had genes necessary for the production of these compounds. Specifically, *Acetobacter pasteurianus* correlated with acetic acid, which is associated with vinegary flavors; *L. kefiranofaciens* correlated with carboxylic acids and ketones associated with cheesy flavors and with esters associated with fruity flavors; *L. mesenteroides* correlated with 2,3-butanedione, which is associated with buttery flavors, and with acetic acid; and *S. cerevisiae* correlated with esters. Sensory analysis revealed that Fr1, a kefir high in *L. mesenteroides*, had a likeable buttery flavor, whereas Ick, a kefir high in *L. kefiranofaciens*, had a less likeable but fruity flavor. Thus, our data suggested a causal relationship between specific taxa and flavor characteristics that was subsequently supported by experimentally manipulating the kefir community. In line with predictions, adding *L. kefiranofaciens* NCFB 2797 to Fr1 resulted in increases in the levels of 2-heptanone and esters, whereas the addition of *L. mesenteroides* 213M0 to Ick resulted in increases in the levels of acetic acid and 2,3-butanediol, a precursor of 2,3-butanedione. However, sensory analysis indicated that these changes were imperceptible and therefore higher inoculum levels might be necessary to change flavor.

On the basis of these results, we predict that the final flavor of kefir can be manipulated by altering the ratio of microbes in the grain. Unfortunately, to date, it has not been possible to artificially reconstruct kefir grains in the laboratory and this might hamper the practical application of our findings. However, we propose that the approach outlined here can be used to accelerate the development of superior multistrain starter cultures to improve the flavor of a variety of fermented foods.

From a systems biology perspective, our work confirms that kefir is suitable as a model microbial community. There are two advantages to using the kefir model, rather than other fermented foods, in this way. First, kefir contains fewer species and so is a simpler environment in which to investigate how microbial communities are formed. Second, kefir is quick and easy to produce, with the fermentation taking just 24 h when it is incubated at room temperature. In addition, others have demonstrated that kefir is a highly culturable system and, indeed, all of the species that were detected at a relative abundance of >1% at 8 and 24 h across the kefirs examined have been isolated previously (39).

Ultimately, Kraken and MetaPhlAn2 showed that the microbial population of kefir was dominated by *L. kefiranofaciens* at 8 h of fermentation. However, between 8 and 24 h, there was a fall in the relative abundance of *L. kefiranofaciens* and *L. mesenteroides* superseded it as the dominant species. The shift from *L. kefiranofaciens* to *L. mesenteroides* is similar to patterns of microbial succession seen in other fermented foods (40, 41). We propose that kefir could be a particularly appropriate model community in which to determine the driving forces behind microbial succession. Early colonizing bacteria in other fermentations have been reported to modify the environment in such a way as to make it more suitable for the growth of other bacteria, thus driving succession (5), and this could explain the observed shift that occurs during kefir fermentation. Our HUMAnN2 results revealed that genes involved in aromatic amino acid biosynthesis were assigned to *L. mesenteroides* but not to *L. kefiranofaciens*. This may be significant because free amino acid analysis showed that there was a significant decrease in the levels of tyrosine in kefir between 8 and 24 h (see Text S1 in the supplemental material). It is possible that its ability to synthesize tyrosine underlies the increased prevalence of *L. mesenteroides*, relative to *L. kefiranofaciens*, in the latter stages of fermentation. Future work will focus on investigating the effect of modifying the levels of tyrosine on the microbiota and volatile-compound profile of kefir. Thus, a “kefir model” has the potential to yield insights into the effects of nutrient availability on microbial succession and metabolite production in other, more complicated, environments.

10.1128/mSystems.00052-16.5 Text S1  Supplemental materials and methods and results. Download Text S1, DOCX file, 0.02 MB. Copyright © 2016 Walsh et al. 2016 Walsh et al. This content is distributed under the terms of the Creative Commons Attribution 4.0 International license.

Finally, we showed that *L. kefiranofaciens* has genes that encode proteins that are considered to be important for probiotic action, including EPS synthesis proteins, bile salt transporters, mucus binding proteins, and bacteriolysins (42, 43). The presence of these genes suggests that the *L. kefiranofaciens* strains present in these kefirs have the potential to survive gastric transit, colonize the gut, and inhibit the growth of pathogens. Indeed, previous studies using mice have shown that *L. kefiranofaciens* protects against enterohemorrhagic *Escherichia coli* infection (44). Further analysis of shotgun metagenomic data from cheese and kimchi indicated that similar genes are present in other fermented foods. Our findings are consistent with previous observations that fermented food-borne microbes can colonize the gut (45) and support designating some fermented foods, like kimchi, “probiotic foods” (31).

In summary, in this study, it has been demonstrated that a combined metagenomic and metabolomic approach can potentially be used to identify the microbes from a particular environment that are responsible for the production of certain metabolites, using the production of flavor compounds during kefir fermentation as a model. Furthermore, we have provided additional evidence of the use of microbial fermentations to provide valuable insights into the dynamics of microbial succession and, in the process, identified genes in *L. kefiranofaciens* that potentially confer important probiotic traits. To conclude, our analyses confirm the value of using kefir as a model microbial community, while also providing a valuable insight into the microbiology of this natural health-promoting beverage.

## MATERIALS AND METHODS

### Kefir fermentations.

Three kefir grains, Fr1, Ick, and UK3, from distinct geographic locations, France, Ireland, and the United Kingdom, respectively, were used for kefir fermentations. The grains were weighed and inoculated in full-fat pasteurized milk at a concentration of 2% (wt/vol) in separate fermentation vessels. The milk was incubated at 25°C for 24 h. A 20-ml volume of milk was collected after 0, 8, or 24 h. In total, there were 15 2% (wt/vol) kefir milk samples: three 0-h samples that were collected immediately before the addition of Fr1, Ick, or UK3; three 8-h samples (one each from Fr1, Ick, and UK3); and nine 24-h samples (one from each of the three replicate fermentations with Fr1, Ick, or UK3). The samples were stored at −20°C until DNA extraction and volatile-compound analysis. Kefir grains were washed with sterile deionized water between fermentations.

Additional fermentations were performed in which milk inoculated with specific kefir grains was supplemented with kefir isolates to assess the consequences of increased levels of these taxa on volatile-compound levels and flavor. Specifically, *L. kefiranofaciens* NCFB 2797 and *L. mesenteroides* 213M0 were grown overnight in 10 ml of de Man, Rogosa, and Sharpe broth; pelleted at 5,444 × *g*; and resuspended in 5 ml of pasteurized milk. *L. kefiranofaciens* NCFB 2797 cells were added to Fr1 milk, and *L. mesenteroides* 213M0 cells were added to Ick milk. Unspiked Fr1 and Ick served as negative controls. As described above, milk was incubated at 25°C for 24 h and the fermentations were carried out in triplicate. A 5-ml volume of milk was collected for volatile-compound analysis, and the samples were stored at −20°C. A 400-ml volume of milk was collected for sensory evaluation, and the samples were stored at −80°C.

### Volatile-compound profiling of kefir by GC-MS.

For volatile-compound analysis of kefir, 1 g of the sample was added to a 20-ml screw-cap solid-phase microextraction (SPME) vial with a silicone/polytetrafluoroethylene septum (Apex Scientific, Maynooth, Ireland) and equilibrated to 75°C for 5 min with pulsed agitation for 5 s at 400 rpm with a GC Sampler 80 (Agilent Technologies Ltd., Little Island, Cork, Ireland). A single 50/30-µm Carboxen-divinylbenzene-polydimethylsiloxane SPME fiber (Agilent Technologies Ltd., Ireland) was used; it was exposed to the headspace above the samples for 20 min at a depth of 1 cm at 75°C. The fiber was retracted and injected into the GC inlet and desorbed for 2 min at 250°C. After injection, the fiber was heated in a bakeout station for 3 min at 270°C to cleanse the fiber. The samples were analyzed in triplicate. Injections were made on an Agilent 7890A GC apparatus with an Agilent DB-5 column (60 m by 0.25 mm by 0.25 µm) with a multipurpose injector with a Merlin microseal (Agilent Technologies Ltd., Ireland). The temperature of the column oven was set at 35°C, held for 0.5 min, increased at 6.5°C·min^−1^ to 230°C, and then increased at 15°C·min^−1^ to 325°C, yielding at total run time of 36.8 min. The carrier gas was helium held at a constant pressure of 23 lb/in^2^. The detector was an Agilent 5975C MSD single-quadrupole mass spectrometer detector (Agilent Technologies Ltd., Ireland). The ion source temperature was 230°C, the interface temperature was set at 280°C, and the MS mode was electronic ionization (−70 V) with the mass range scanned between 35 and 250 atomic mass units. Compounds were identified by mass spectrum comparisons to the National Institute of Standards and Technology 2011 mass spectral library, the automated mass spectral deconvolution and identification system, and an in-house library created in TargetView software (Markes International, Llantrisant, United Kingdom) with target and qualifier ions and linear retention indices for each compound. Autotuning of the GC-MS system was carried out prior to the analysis to ensure optimal GC-MS performance. A set of external standards was also run at the start and end of the sample set, and abundances were compared to known amounts to ensure that both the SPME extraction and MS detection were performing within specifications.

Volatile-compound profiling of spiked and unspiked kefir samples was done by a slightly modified GC-MS protocol (see Text S1 in the supplemental material).

### Sensory analysis of spiked and nonspiked kefir.

Twenty-five naive assessors were recruited for sensory acceptance evaluation, and 10 trained assessors were recruited for RDA. Analysis of variance–partial least-squares regression (APLSR) was used to process the results of the sensory acceptance evaluation test and RDA with Unscrambler software version 10.3. See Text S1 in the supplemental material for a more in-depth description of the sensory analysis methods used.

### Total DNA extraction from kefir (milks and grains).

DNA was extracted from 15 ml of kefir milk as follows. Milk was centrifuged at 5,444 × *g* for 30 min at 4°C to pellet the microbial cells in the liquid. The cell pellet was resuspended in 200 µl of PowerBead solution from the PowerSoil DNA Isolation kit (Cambio, Cambridge, United Kingdom). The resuspended cells were transferred to a PowerBead tube (Cambio, Cambridge, United Kingdom). A 90-µl volume of 50 mg/ml lysozyme (Sigma-Aldrich, Dublin, Ireland) and 50 µl of 100 U/ml mutanolysin (Sigma-Aldrich, Dublin, Ireland) were added, and the sample was incubated at 60°C for 15 min. A 28-µl volume of proteinase K (Sigma-Aldrich, Dublin, Ireland) was added, and the sample was incubated at 60°C for a further 15 min. DNA was then purified from the sample by the standard PowerSoil DNA Isolation kit protocol (Cambio, Cambridge, United Kingdom). Total DNA was also extracted from each of the three grains. Fragments of 50 mg were removed from different sites on each of the grains and added to separate PowerBead tubes (Cambio, Cambridge, United Kingdom). The grain fragments were homogenized by shaking the PowerBead tube on the TissueLyser II (Qiagen, West Sussex, United Kingdom) at 20 Hz for 10 min. Following homogenization, DNA was purified from the sample by the method outlined above. Total DNA was initially quantified and qualified by gel electrophoresis and the NanoDrop 1000 (BioSciences, Dublin, Ireland) before more accurate quantification with the Qubit High Sensitivity DNA assay (BioSciences, Dublin, Ireland). Bacterial and fungal abundances were determined by qPCR by the protocol described by Fouhy et al. (46) and the Femto Fungal DNA Quantification kit (Cambridge Biosciences, United Kingdom), respectively.

### Amplicon sequencing.

16S rRNA gene libraries were prepared from extracted DNA by the 16S Metagenomic Sequencing Library Preparation protocol from Illumina (47). ITS gene libraries were prepared for the samples with a modified version of the 16S rRNA gene extraction protocol. Briefly, the initial genomic DNA amplification was performed with primers specific to the ITS1-ITS2 region of the ITS gene (48), but they were modified to incorporate the Illumina overhang adaptor (i.e., ITSF1 primer 5′ TCGTCGGCAGCGTCAGATGTGTATAAGAGACAGCTTGGTCATTTAGAGGAAGTAA 3′ and ITS2 primer 5′ GTCTCGTGGGCTCGGAGATGTGTATAAGAGACAGGCTGCGTTCTTCATCGATGC 3′). After amplification of the ITS1-ITS2 region, PCR products were treated as described in the Illumina protocol. Samples were sequenced on the Illumina MiSeq in the Teagasc sequencing facility, with a 2 × 250 cycle V2 kit, in accordance with standard Illumina sequencing protocols.

### Whole-metagenome shotgun sequencing.

Whole-metagenome shotgun libraries were prepared in accordance with the Nextera XT DNA Library Preparation Guide from Illumina (47). Samples were sequenced on the Illumina MiSeq sequencing platform in the Teagasc sequencing facility, with a 2 × 300 cycle V3 kit, in accordance with standard Illumina sequencing protocols.

### Bioinformatic analysis.

16S rRNA gene sequencing data were processed with the pipeline described by Fouhy et al. (49). Briefly, sequences were quality checked, clustered into operational taxonomic units, and aligned and diversity (both alpha and beta) was calculated with a combination of the Qiime (1.8.0) (50) and USearch (v7-64bit) (51) pipelines. Taxonomy was assigned with a BLAST search (52) against SILVA SSURef database release 1 (53). ITS gene sequencing data were processed with a slightly modified pipeline. Taxonomy was assigned by using a BLAST search against the ITSoneDB database (54). Raw reads from whole-metagenome shotgun sequencing were filtered on the basis of quality and quantity and trimmed to 200 bp with a combination of Picardtools (https://github.com/broadinstitute/picard) and SAMtools (55). Subsequently, function was assigned to reads with the HUMAnN2 suite of tools (56), which assigned function based on the ChocoPhlan databases and genes based on UniRef (57). The HUMAnN2 gene abundance table was regrouped by a mapping of MetaCyc pathways and a mapping of GO terms for amino acid, carbohydrate, and lipid metabolism. MetaPhlAn2 and Kraken were used to profile changes in the microbial composition of kefir milk at the species level (24, 25).

### Statistical analysis of metagenomic and metabolomic data.

Statistical analysis was done with R-3.2.2 (58) and LEfSe (28). The R packages ggplot2 and gplots and the cladogram generator Graphlan (59) were used for data visualization.

### Accession number(s).

Sequence data have been deposited in the European Nucleotide Archive (ENA) under the project accession number PRJEB15432.
