# Correction for Walsh et al., Microbial Succession and Flavor Production in the Fermented Dairy Beverage Kefir

**DOI:** 10.1128/mSystems.00003-17

**Published:** 2017-02-21

**Authors:** Aaron M. Walsh, Fiona Crispie, Kieran Kilcawley, Orla O’Sullivan, Maurice G. O’Sullivan, Marcus J. Claesson, Paul D. Cotter

**Affiliations:** a Teagasc Food Research Centre, Moorepark, Fermoy, Co. Cork, Ireland; b APC Microbiome Institute, University College Cork, Co. Cork, Ireland; c Microbiology Department, University College Cork, Co. Cork, Ireland; d School of Food and Nutritional Sciences, University College Cork, Co. Cork, Ireland

## AUTHOR CORRECTION

Volume 1, no. 5, 2016, https://doi.org/10.1128/mSystems.00052-16. In this paper, the strain referred to as *Leuconostoc mesenteroides* 213M0 was erroneously labeled. The strain used was isolated from kefir, and its 16S rRNA gene was sequenced by Sanger sequencing. Subsequent sequence alignment indicated that the strain used was most closely related to *L. mesenteroides* 213M0, which was isolated by K. Arakawa et al. (Anim Sci J 87:449–456, 2015, https://doi.org/10.1111/asj.12445). We have since provided the strain used with a unique designation, *Leuconostoc mesenteroides* DPC 7047, and it has been deposited in the DPC Culture Collection in Teagasc Moorepark, Fermoy, County Cork, Ireland.

