# Supplementary material for: Microbial Succession and Flavor Production in the Fermented Dairy Beverage Kefir
Source: mSystems. 2016 Oct 4;1(5):e00052-16. doi: 10.1128/mSystems.00052-16 (PMC5080400; doi:10.1128/mSystems.00052-16)
Supplement: Figure S1 [file sys005162055sf1.pdf]

A

B

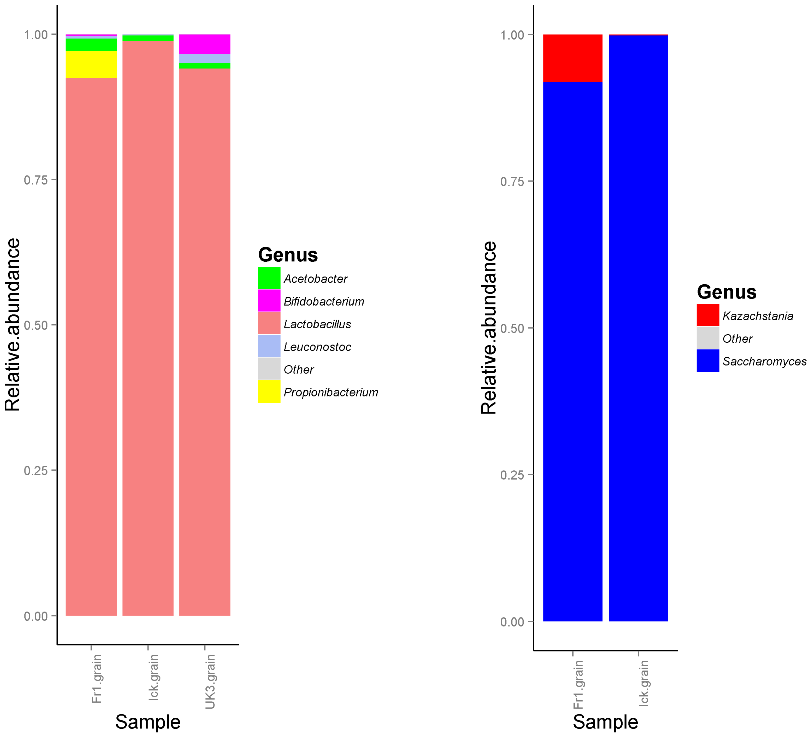

Figure S1. The (a) bacterial and (b) fungal composition of kefir grains, as determined by amplicon sequencing. Note that we were unable to generate an ITS amplicon for the UK3 sample.
