# Supplementary material for: Microbial Succession and Flavor Production in the Fermented Dairy Beverage Kefir
Source: mSystems. 2016 Oct 4;1(5):e00052-16. doi: 10.1128/mSystems.00052-16 (PMC5080400; doi:10.1128/mSystems.00052-16)
Supplement: Figure S2 [file sys005162055sf2.docx]

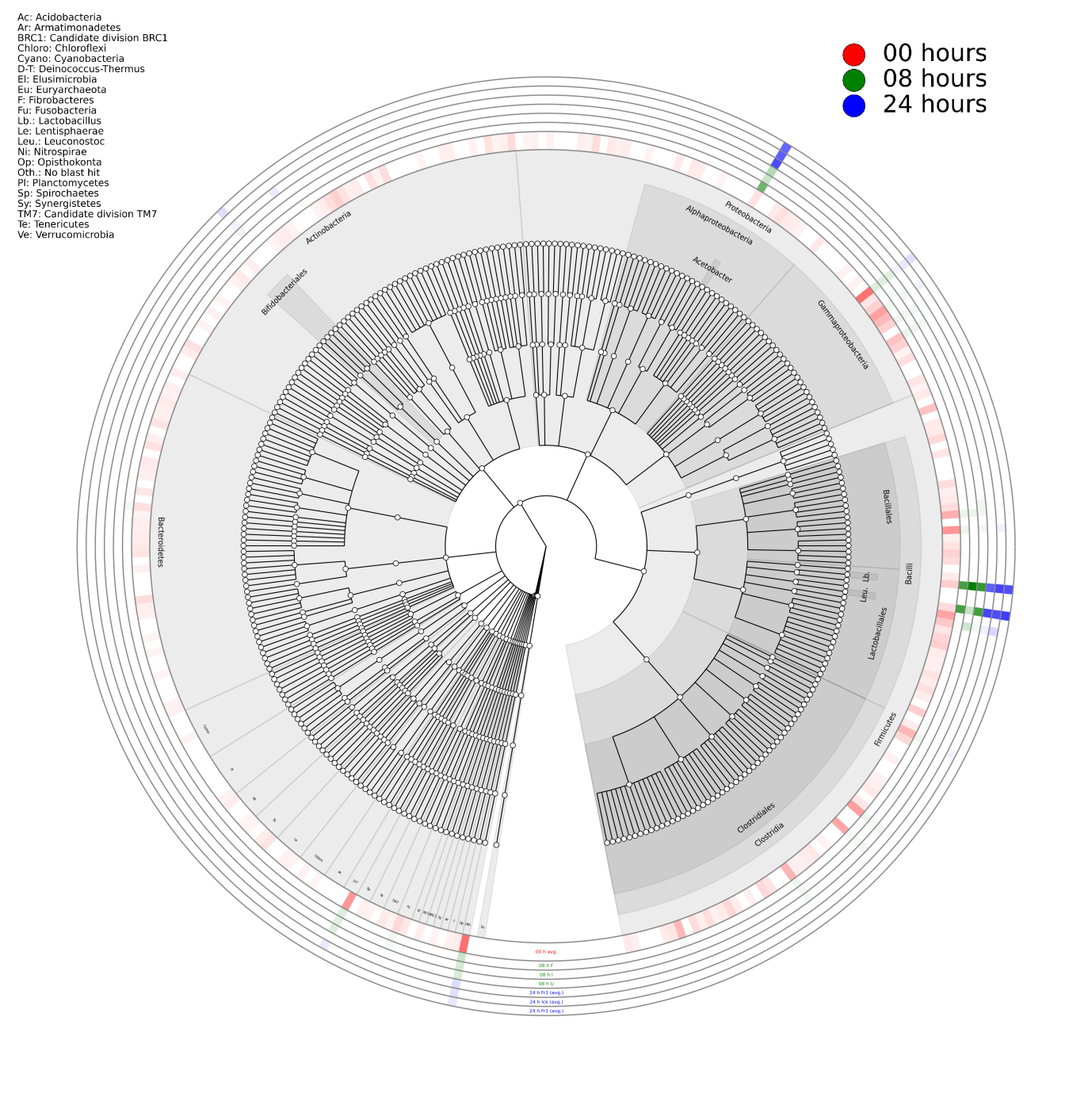


**Figure S2. Cladogram presenting the bacterial diversity of kefir samples at 0, 8 and 24 hours of fermentation, as determined by 16S rRNA gene sequencing.** The colour intensities in the outer rings indicate the average relative abundance of each bacterial genus in Fr1, Ick and UK3 at each stage.
