# Supplementary material for: Microbial Succession and Flavor Production in the Fermented Dairy Beverage Kefir
Source: mSystems. 2016 Oct 4;1(5):e00052-16. doi: 10.1128/mSystems.00052-16 (PMC5080400; doi:10.1128/mSystems.00052-16)
Supplement: Table S1 [file sys005162055st1.pdf]

Table S1. Absolute abundances of bacteria and fungi in kefir samples after 0, 8 and 24 hours of fermentation, as determined by quantitative PCR (qPCR) measurements.

Sample	Total fungi (ng of fungal DNA)	Total bacteria (copies of 16S rRNA gene)
Milk 0 h	0.0016	1.78E+05
Fr1 08 h	0.1386	7.64E+07
Fr1 24 h	0.2179	2.49E+08
Ick 08 h	0.0542	2.62E+08
Ick 24 h	0.0972	1.63E+08
UK3 08 h	0.0896	7.86E+07
Uk3 24 h	0.3933	4.22E+08
