# Supplementary figures and images for: Microbial Succession and Flavor Production in the Fermented Dairy Beverage Kefir
Source: mSystems. 2016 Oct 4;1(5):e00052-16. doi: 10.1128/mSystems.00052-16 (PMC5080400; doi:10.1128/mSystems.00052-16)

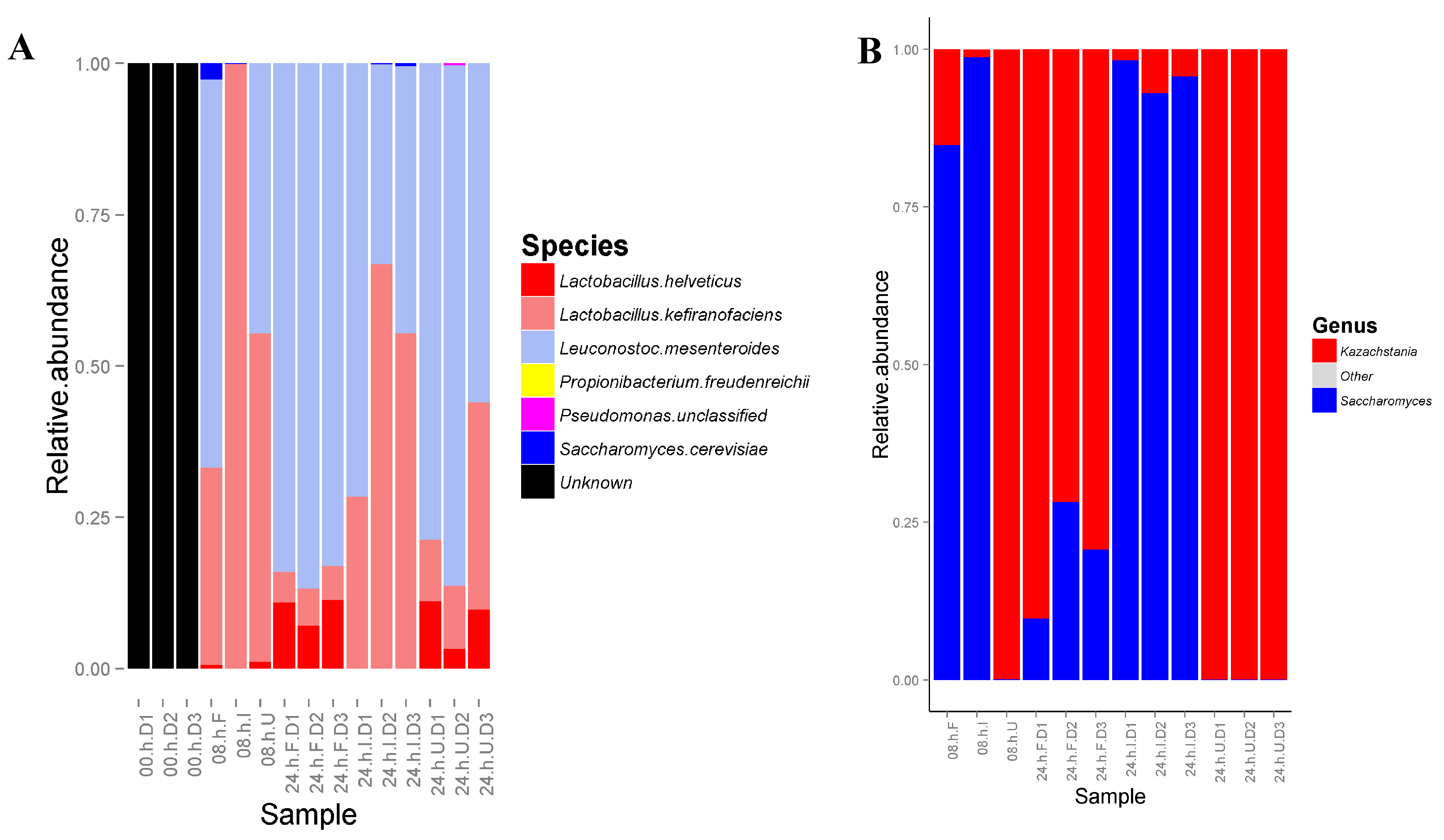

Supplement: Figure S3 [file sys005162055sf3.tif]

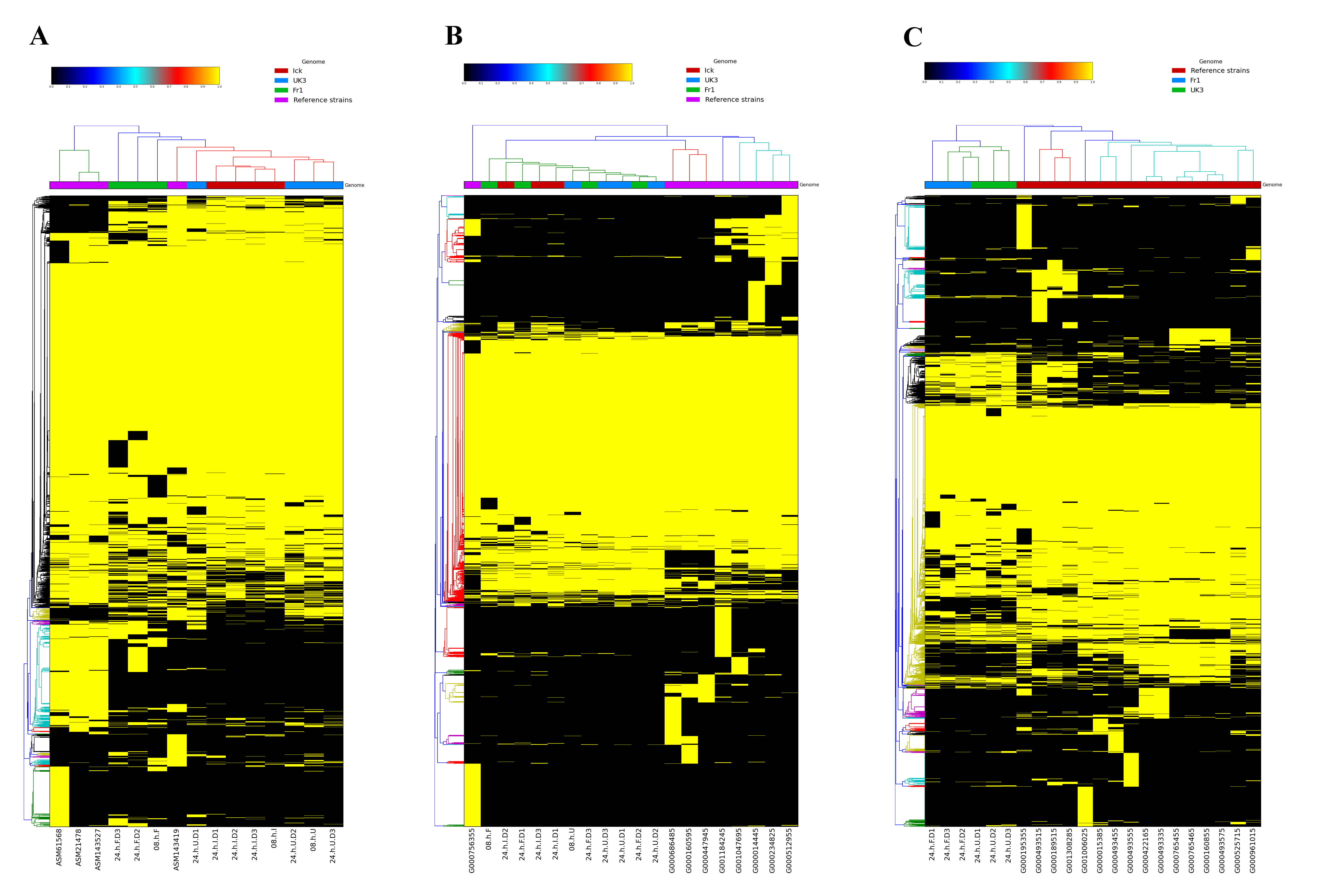

Supplement: Figure S4 [file sys005162055sf4.tif]
