# Supplementary material for: Microbial Succession and Flavor Production in the Fermented Dairy Beverage Kefir
Source: mSystems. 2016 Oct 4;1(5):e00052-16. doi: 10.1128/mSystems.00052-16 (PMC5080400; doi:10.1128/mSystems.00052-16)
Supplement: Figure S5 [file sys005162055sf5.pdf]

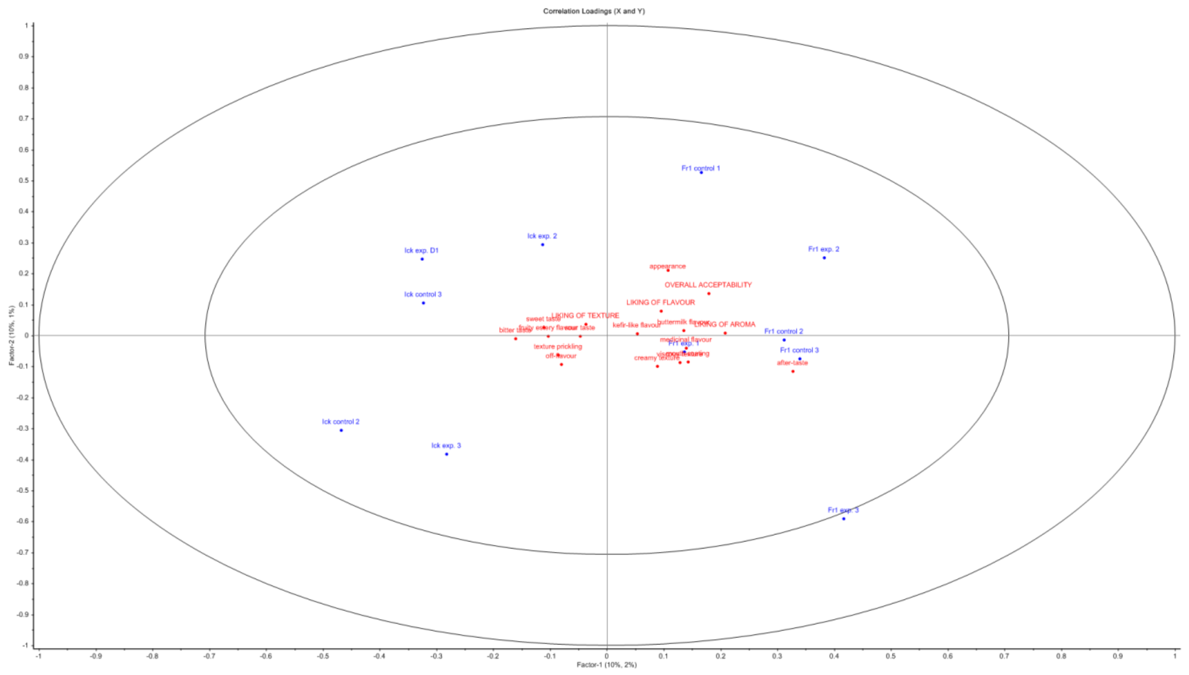

Figure S5. ANOVA-Partial Least Squares Regression (ASLPR, PCs 1-2) plot for spiked and non-spiked kefir samples, presented are Sensory Acceptance and Ranking Descriptive Analysis data.
