# Supplementary material for: Microbial Succession and Flavor Production in the Fermented Dairy Beverage Kefir
Source: mSystems. 2016 Oct 4;1(5):e00052-16. doi: 10.1128/mSystems.00052-16 (PMC5080400; doi:10.1128/mSystems.00052-16)
Supplement: Table S3 [file sys005162055st3.docx]

Table S3. Correlations between the relative abundances of microbial genera and the levels of volatile compounds

| **Genus** | **Compound(s)** | **R-value** | **Uncorrected p-value** |
| --- | --- | --- | --- |
| **Acetobacter** | Acetic acid | 0.76 | <0.01 |
|  | 2-methyl-1-butanol | 0.65 | 0.01 |
|  | 2,3-butanedione | 0.67 | <0.01 |
| **Kazachstania** | Acetic acid | 0.52 | 0.05 |
|  | 2-methyl-1-butanol | 0.53 | 0.04 |
|  | 2,3-butanedione | 0.85 | <0.01 |
|  | 2,3-pentanedione | 0.68 | <0.01 |
|  | 2,3-hexanedione | 0.72 | <0.01 |
| **Lactobacillus** | Carboxylic acids | 0.6 | 0.02 |
|  | Esters | 0.59 | 0.02 |
|  | 3-methyl-1-butanol | 0.58 | 0.02 |
| **Leuconostoc** | 2,3-butanedione | 0.68 | 0.005 |
| **Saccharomyces** | Carboxylic acids | 0.71 | <0.01 |
|  | Esters | 0.78 | <0.01 |
