# Supplementary material for: Microbial Succession and Flavor Production in the Fermented Dairy Beverage Kefir
Source: mSystems. 2016 Oct 4;1(5):e00052-16. doi: 10.1128/mSystems.00052-16 (PMC5080400; doi:10.1128/mSystems.00052-16)
Supplement: Text S1 [file sys005162055s1.docx]

**Supplemental materials and methods**

**Volatile profiling of spiked kefir by GCMS.** 2 g of sample was added to 20 ml screw capped SPME vial and equilibrated to 40 °C for 10 mins with pulsed agitation of 5 sec at 500 rpm.  The samples were analysed in triplicate.  Sample introduction was accomplished using a CTC Analytics CombiPalAutosampler.

  A single 50/30 µm Carboxen^TM^/divinylbenzene/polydimethylsiloxane (DVB/CAR/PDMS) fiber was used.  The SPME fiber was exposed to the headspace above the samples for 20 min at depth of 1 cm at 40°C.  The fiber was retracted and injected into the GC inlet and desorbed for 2 min at 250°C.  Injections were made on an Shimadzu 2010 Plus GC with an Agilent DB-5 (60 m x 0.25 mm x 0.25 μm) column using a split/splitless injector in splitless mode with a merlin microseal.  The temperature of the column oven was set at 35°C, held for 0.5 min, increased at 6.5°C/min to 230°C then increased at 15°C/min to 320°C, yielding at total GC run time of 41.5 min.  The carrier gas was helium held at a constant pressure of 23 psi.  The detector was a Shimadzu TQ8030 mass spectrometer detector, ran in single quad mode.  The ion source temperature was 220°C and the interface temperature were set at 280°C and the MS mode was electronic ionization (-70 v) with the mass range scanned between 35 and 250 amu.  Compounds were identified using mass spectra comparisons to the NIST 2014 mass spectral library and in-house library created in Chem Solutions software (Shimadzu, Japan) with target and qualifier ions and linear retention indices for each compound.  Final data processing was undertaken using TargetView deconvolution software (Markes International Ltd, UK).  An auto-tune of the GCMS was carried out prior to the analysis to ensure optimal GCMS performance.  A set of external standards was ran at the start and end of the sample set and abundances were compared to known amounts to ensure that both the SPME extraction and MS detection was performing within specification.

**Sensory acceptance evaluation of spiked and non-spiked kefir milks.** Twenty five naïve assessors were recruited in University College Cork, Ireland for sensory acceptance evaluation of spiked and non-spiked kefir milks. Age range of assessors was 21-48 years old. The selection criteria for assessors were availability and motivation to participate on all days of the experiment. Assessors used sensory Hedonic descriptors (Table SX) on 11 different kefir samples (Table SX). Sensory analysis was carried out in panel booths conforming to international standards (ISO 8589:2007). All samples were stored at -20˚C until required. Samples were then held at refrigeration temperatures overnight (4ºC), before monadic presentation to the consumer panel at ambient temperatures (21°C) and coded with a randomly selected 3 digit code. A maximum of six samples were presented at each session. Each assessor was provided with deionised water and instructed to cleanse their palates between tastings asked to assess the attributes, according to a ten-point scale. The order of the presentation of all test samples was randomized to prevent first order and carryover effects.

**Ranking descriptive analysis (RDA) of spiked and non-spiked kefir milks.** Ten panellists were recruited in University College Cork, Ireland. Age range of assessors was 25-45 years old. Selection criteria for panellists were availability and motivation to participate on all days of the experiment and that they were familiar with kefir as a product. All panellists had participated in dairy descriptive profiles in the past and were well versed in the sensory experimental protocol. Panellists were trained using the sensory Intensity descriptors (Table SX). Ranking Descriptive analysis (RDA) [[2](#_ENREF_2), [3](#_ENREF_3)] was carried out in panel booths conforming to international standards (ISO 8589:2007) on the 11 Kefir samples to be tested. All samples were stored at -20˚C until required. Samples were then held at refrigeration temperatures overnight (4ºC), before presentation to the panel at ambient temperatures (21°C) and coded with a randomly selected 3 digit code. The Kefir samples were immediately served to panellists simultaneously in separate sessions for Fr1 and Ick variants. Each assessor was provided with deionised water and instructed to cleanse their palates between tastings. Additionally, each assessor was presented with samples and asked to rank the intensity of the attributes, according a 10 cm line scale ranging from 0 (none) at the left to 10 (extreme) at the right and rating subsequently scored in cm from left (Table SX). The order of the presentation of all test samples was randomized to prevent first order and carryover effects.

**Statistical analysis of sensory analysis data.** For evaluating the results of the RDA and the sensory acceptance test, ANOVA-Partial Least Squares regression (APLSR) was used to process the data accumulated using Unscrambler software version10.3. The X-matrix was designed as 0/1 variables for sample and the Y-matrix sensory variables.

**Free amino acid analysis.** The aromatic amino acids phenylalanine and tyrosine were quantified in the milk samples using the method described by McDermott *et al.* [[1](#_ENREF_1)].

**Supplemental results**

**Sequencing results.** 16S rRNA gene sequencing generated 5,545,825 reads in total and an average of 210,136 reads per sample, while ITS gene sequencing generated 3,498,902 reads in total and an average of 291,567 reads per sample. Whole metagenome sequencing generated a total of 22,983,010 reads and an average of 1,209,632 reads per sample.

**Free amino acid analysis results.** Free amino acid analysis showed that the levels of phenylalanine increased from 0.63 to 0.98 nmol/ml between 8 and 24 hours, but the Wilcoxon signed rank test indicated that this increase was not statistically significant (p=0.064). In contrast, the levels of tyrosine decreased from 4.18 to 1.42 nmol/ml between 8 and 24 hours, and the Wilcoxon signed rank test indicated that this decrease was statistically significant (p=0.018).

**References**

1. McDermott, A., et al., *Prediction of individual milk proteins including free amino acids in bovine milk using mid-infrared spectroscopy and their correlations with milk processing characteristics.* J Dairy Sci, 2016.

2. Richter, V.B., et al., *Proposing a ranking descriptive sensory method.* Food Quality and Preference, 2010. **21**(6): p. 611-620.

3. Dairou, V. and J.M. Sieffermann, *A comparison of 14 jams characterized by conventional profile and a quick original method, the flash profile.* Journal of food science, 2002. **67**(2): p. 826-834.
